# You've Gotten Under my Skin: How to Make a Simple, Non-Perishable, Low-Cost Soft Tissue Infection Ultrasound Simulator

**DOI:** 10.24908/pocusj.v10i01.18407

**Published:** 2025-04-15

**Authors:** John Barrett, Christy Moore, Jeffrey A. Kramer, Nova Panebianco

**Affiliations:** 1 Department of Emergency Medicine, Penn Medicine and University of Pennsylvania School of Nursing, Philadelphia, PA, USA; 2 Department of Emergency Medicine, Perelman School of Medicine, University of Pennsylvania, Philadelphia, PA, USA; 3 Department of Emergency Medicine, Division of Emergency Ultrasound, Penn Presbyterian Medical Center, Philadelphia, PA, USA

**Keywords:** abscess, cellulitis, simulation, phantom, trainer, POCUS

## Abstract

We describe how to make an ultrasound compatible, low-cost, non-perishable, durable skin and soft tissue infection (SSTI) phantom model that simulates multiple pathologies including abscess and necrotizing fasciitis. The SSTI simulator has an extended shelf-life, can be recreated, and can serve as a needle aspiration simulator.

## Introduction

Skin and soft tissue infections (SSTI) are prevalent in the United States with over 14 million cases reported annually [1]. In emergency departments, it is important for providers to differentiate between a drainable abscess and cellulitis. Point of care ultrasound (POCUS) has proven to be an effective tool, influencing management in approximately 50% of suspected soft tissue infections [2]. Its sensitivity and specificity in adults are 98.7% and 91%, respectively [3].

A notable impediment to POCUS training for SSTI is the high cost associated with commercially available simulators, which can exceed $1,200. Additionally, during a training session, identifying standardized patients with SSTI pathology is unrealistic. Developing homemade low-cost high-fidelity simulators with realistic SSTI findings is challenging. While methods for crafting SSTI simulators is documented in the literature, the majority involve using food items [4]. Our objective was to devise an ultrasound compatible, non-perishable, reusable alternative. This paper outlines the creation of a SSTI ultrasound phantom simulator housed in an 8” x 5” x 2.5” sized portable container.

## Materials and Methods

### Base Layer

**Figure 1.**
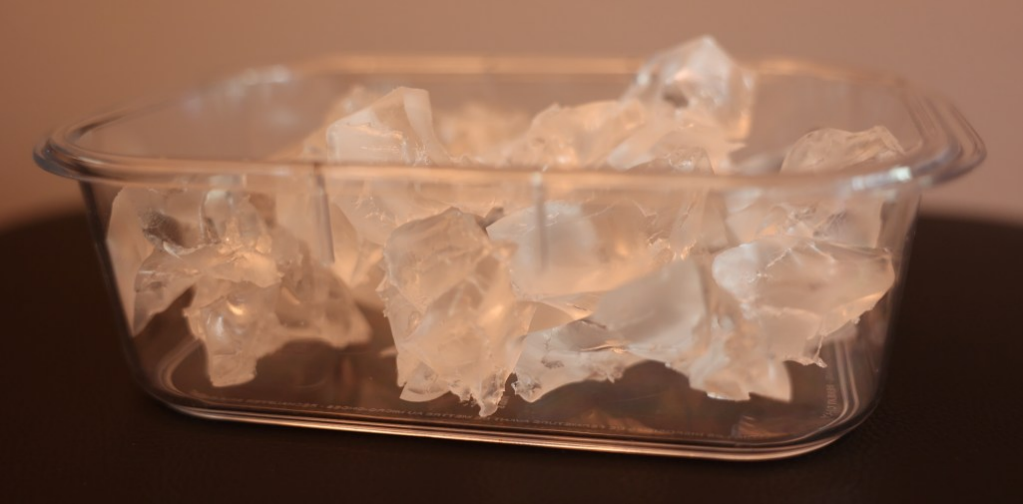
295 grams of ballistics gel used as base layer.

**Figure 2.**
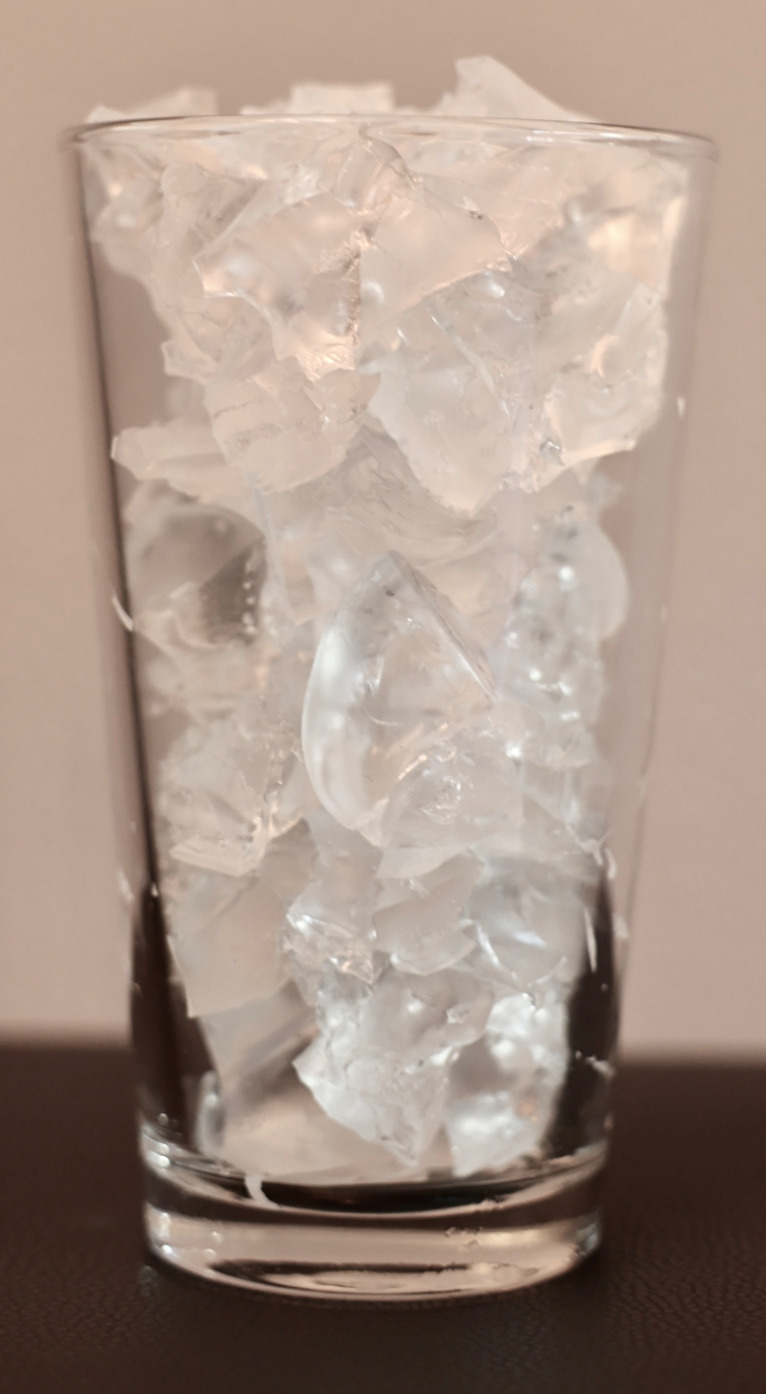
200 grams of ballistics gel used as covering layer.

The SSTI simulator was created in three stages – base, simulated pathology (SSTI), and covering layers. Initially, 295 grams of Clear Ballistics (Greenville, SC) 10% ballistic gel were melted in the oven at 205°F in a Rubbermaid® BrillianceTM 3.2 cup plastic container (Figure 1). Simultaneously, another 200 grams of ballistics gel were melted in a separate pint glass for the covering layer (Figure 2). This process took around 3-4 hours to ensure all air bubbles dissipated.

**Figure 3.**
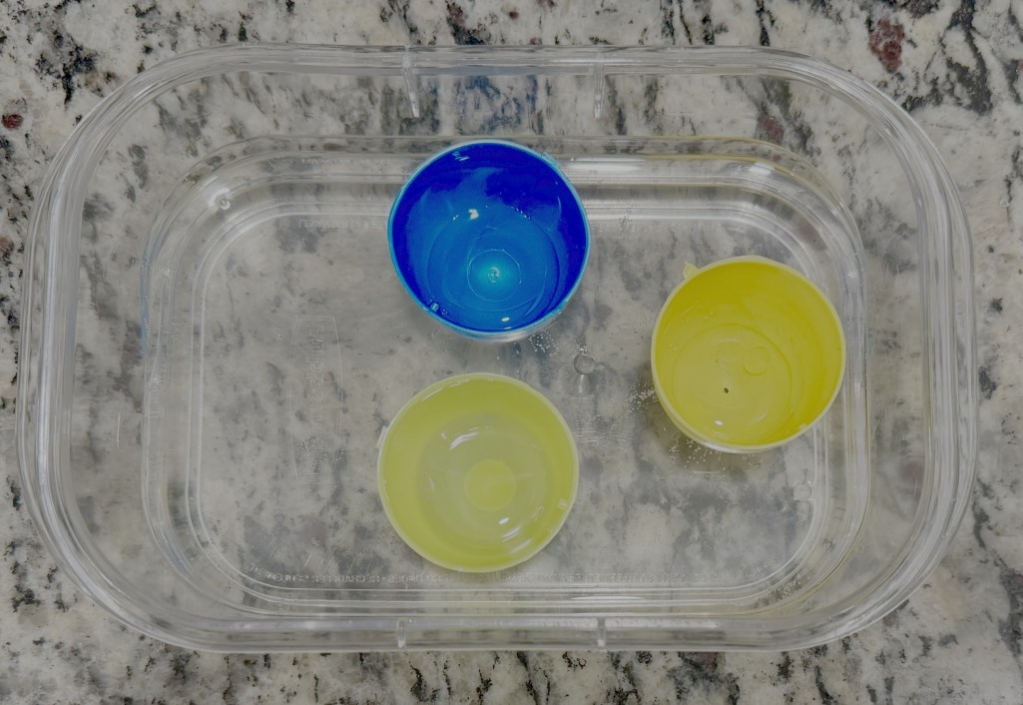

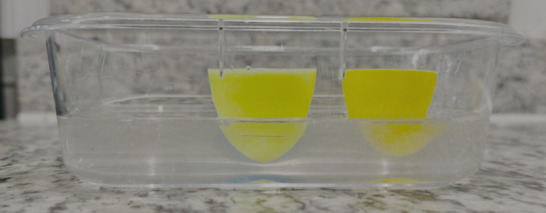
Plastic eggs placed in the base layer one minute after removal from oven.

The container was removed from the oven and allowed to set for one minute. Next, three plastic egg tops were placed on the semi-solid base and filled with 4-5 mL of water to ensure proper depth in the base layer and allowed to set for one hour (Figure 3). After the base layer cooled, the plastic egg tops were removed.

### SSTI Layer

**Figure 4.**
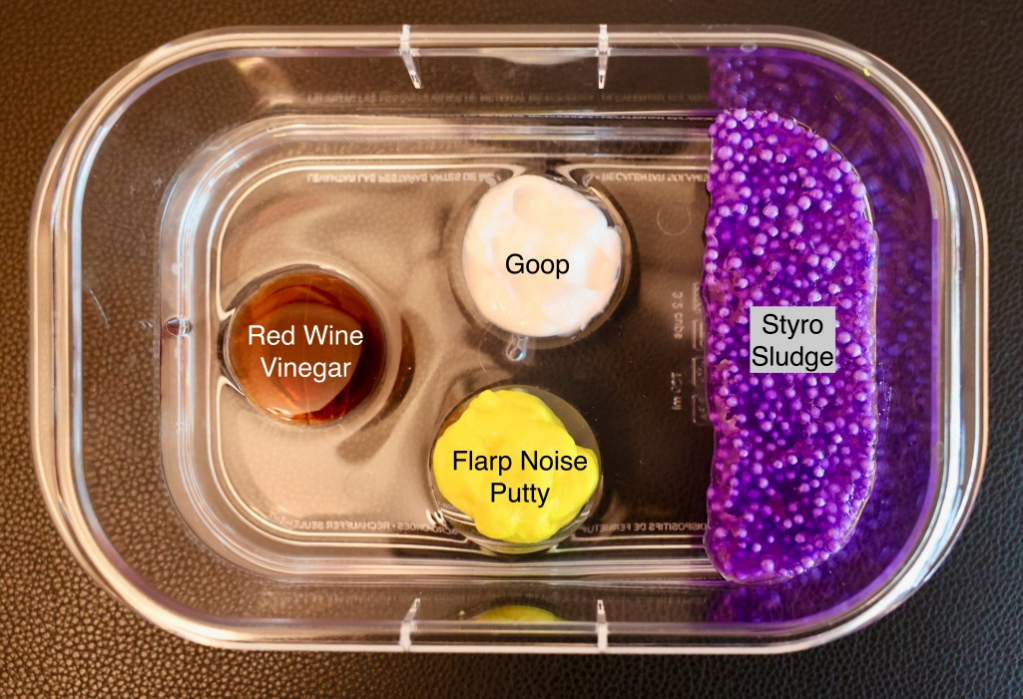

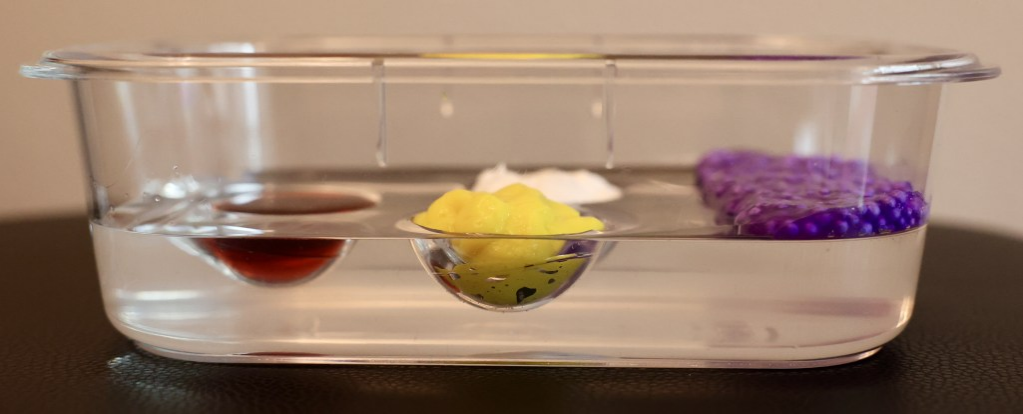
Abscess base layer with labeled contents.

The SSTI layer utilized diverse materials to simulate various echotextures including: 8 grams of Ja-Ru® Flarp Noise Putty® (Jacksonville, FL), 8 mL of Goop® hand cleaner (St. Louis, MO), and 8 mL of red wine vinegar. These materials were separately placed into the prepared molds. We then flattened 13 grams of Ja-Ru® Styro Sludge to simulate necrotizing fasciitis on one end of the base layer (Figure 4). These materials were chosen after extensive testing of over 30 products that were easy to obtain locally and available broadly across the United States.

### Covering Layer

**Figure 5.**
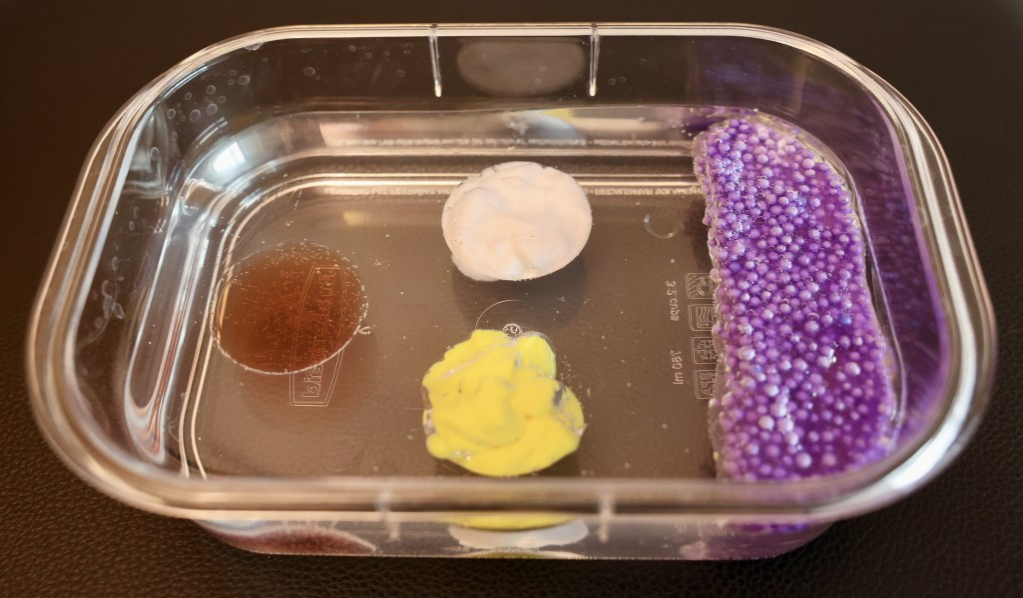

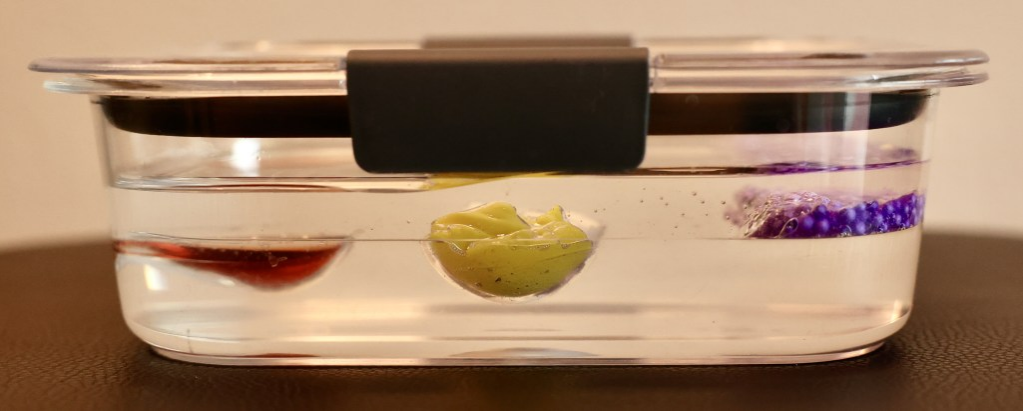
Completed abscess simulator.

The melted gel from the pint glass was then carefully poured to form the covering layer, ensuring minimal disturbances to the SSTI formations. We found that keeping the pint glass as close to the plastic container limited the number of air bubbles in the final product. Additionally, a small amount of vinegar tended to spill to the side while pouring the covering layer. We used a syringe to remove any excess vinegar. The completed simulator required one additional hour to fully set (Figure 5).

## Results

**Figure 6.**
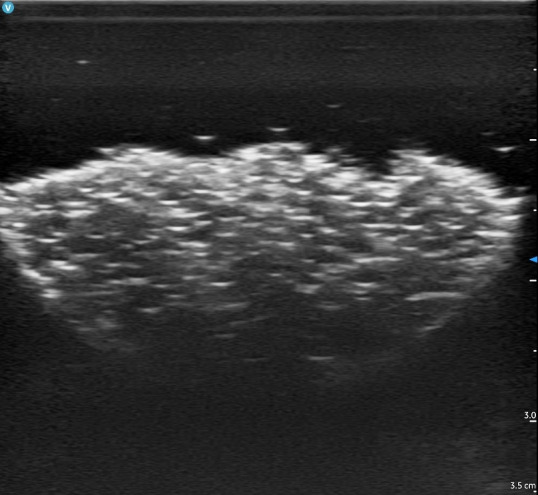
Abscess simulation using Goop hand cleaner imaged by point of care ultrasound (POCUS).

**Figure 7.**
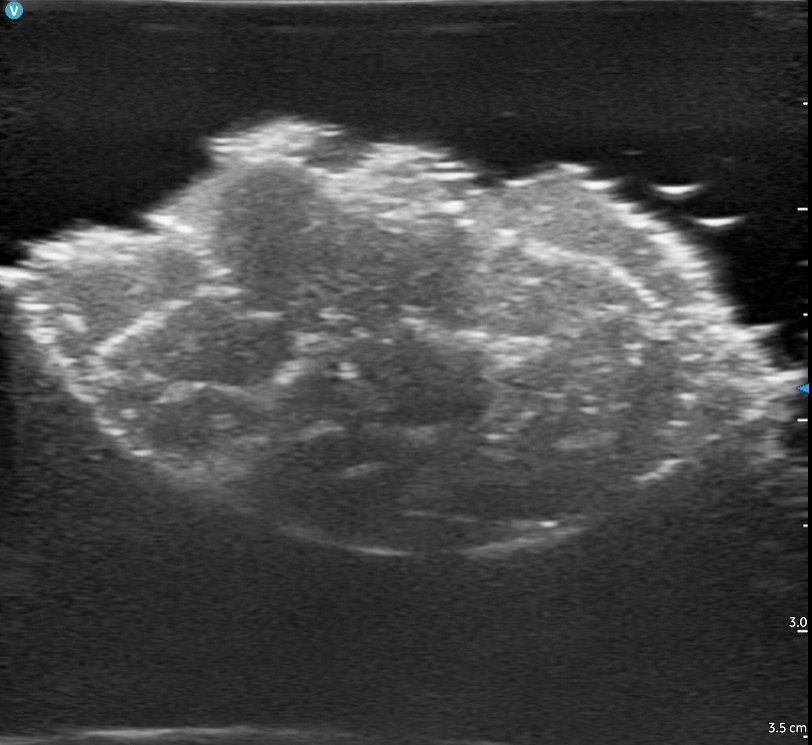
Abscess simulation using Flarp Noise Putty imaged by point of care ultrasound (POCUS).

**Figure 8.**
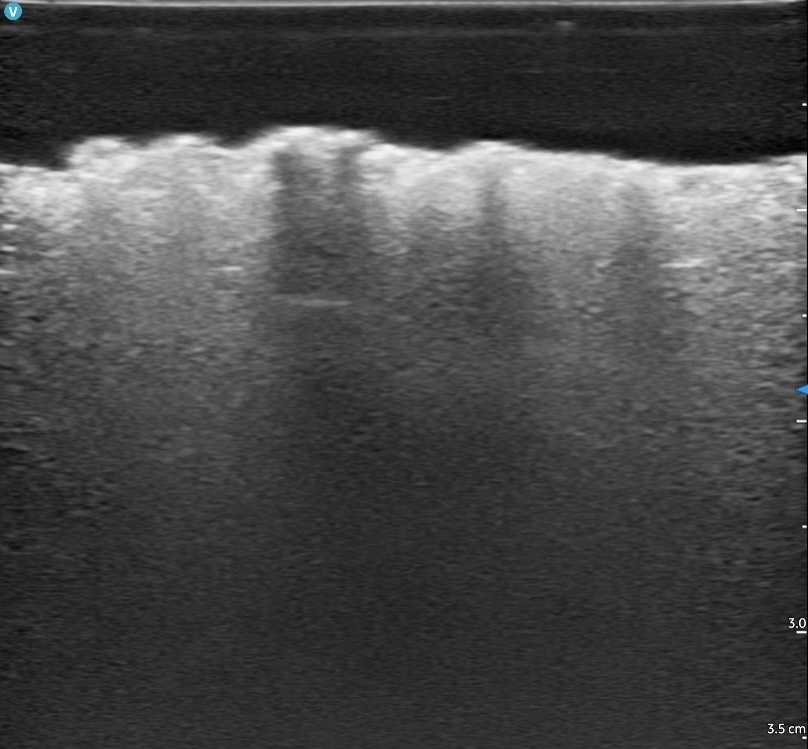
Necrotizing fasciitis simulator using Styro Sludge imaged by point of care ultrasound (POCUS).

**Figure 9.**
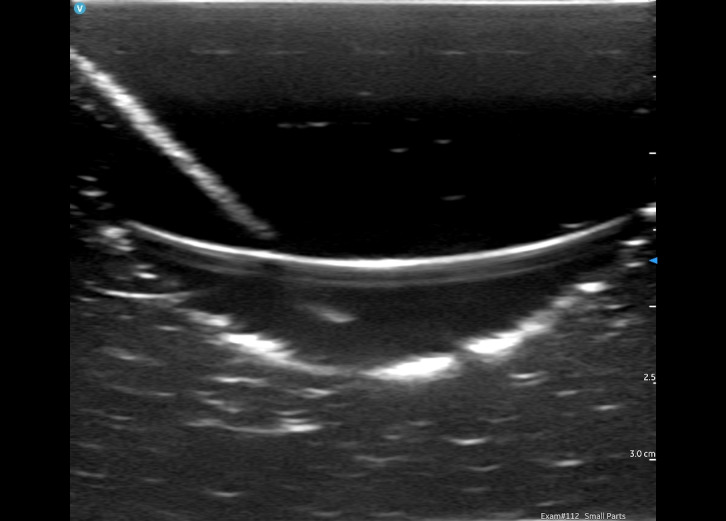
Necrotizing fasciitis simulator using Styro Sludge imaged by point of care ultrasound (POCUS).

The culmination of our efforts yielded a robust, non-perishable ultrasound simulator capable of replicating various SSTI echotextures within a single container. The application of Goop hand cleaner resulted in a heterogeneous echotexture devoid of the loculations typically observed in abscesses (Figure 6). Conversely, the use of flarp noise putty generated echotextures featuring loculations (Figure 7). Styro sludge demonstrated the production of multiple ring-down artifacts, effectively simulating air and necrotizing fasciitis (Figure 8). Notably, red-wine vinegar produced a hypoechoic and homogeneous echotexture with the added functionality that the fluid could be used for ultrasound-guided needle aspiration (the other materials were too viscous to be aspirated with a standard 18-gram needle) (Figure 9).

## Discussion

POCUS proves invaluable in distinguishing various types of SSTIs. However, accessing training is hindered by the prohibitive cost of commercial simulators and lack of standardized patient models with pathologic findings. Our SSTI simulator, priced under $75 if made as a single phantom model, offers an affordable alternative. We made 10 simulators which brought down the cost to $20 per unit. All materials were locally sourced from Walmart® or Dollar General®, except for the ballistics gel which is available online. Please refer to the appendix for a detailed supply list with associated costs.

The SSTI simulator is portable, weighing under two pounds, and occupies minimal space which makes it ideal for educational settings at various levels, including conferences and workshops. Its durability allows for repeated use without significant wear. The abscess model is aesthetically appealing and odorless, featuring a rubber-like texture that leaves no residue during handling. Importantly, the use of non-animal-derived materials mitigates the risk of offending individuals with religious or personal beliefs against the use of animal products for training purposes, while also eliminating the inherent risk of bacterial transmission in a training setting.

Another advantage of this simulator is its ability to simulate four different types of SSTI echotextures, potentially facilitating a more comprehensive understanding of the sonographic findings of various SSTI. Moreover, it provides opportunity for simulating incision and drainage procedures. The incorporation of red-wine vinegar provided good haptic feedback akin to a genuine abscess. However, it was noted that each aspiration left a needle track mark in the ballistics gel which could limit the durability and fidelity of the SSTI simulator over time. While most linear probes have a narrow beam width that enables the avoidance of previous aspiration sites, multiple uses may necessitate remelting of the gel. Nonetheless, this SSTI simulator is designed for remelting, which makes it even more cost-effective after the initial cost of purchasing materials. Of note, the materials used for each abscess can be removed, and the gel can be cleaned with water.

In conclusion, this SSTI simulator offers a practical, affordable, and effective alternative to expensive commercial models, enhancing training in diagnosing and managing SSTIs using POCUS. Its ability to simulate various echotextures and facilitate realistic procedural practice makes it invaluable. The use of non-perishable materials ensures durability and cost-effectiveness, while its portability suits diverse educational settings. Despite minor issues like needle track marks, the model's design allows for remelting and cleaning, ensuring repeated use. Overall, this SSTI simulator represents an innovative approach to POCUS education, improving access to essential training tools and ultimately enhancing patient care.
